# Association between the childhood rearing environment and general and specific psychopathology factors in middle adulthood: a Swedish National High-Risk Home-Reared versus Adopted-Away Sibling Comparison Study

**DOI:** 10.1038/s41380-025-02979-1

**Published:** 2025-04-01

**Authors:** Mengping Zhou, Henrik Larsson, Brian M. D’Onofrio, Mikael Landén, Paul Lichtenstein, Erik Pettersson

**Affiliations:** 1 Department of Medical Epidemiology and Biostatistics, Karolinska Institutet, Stockholm, Sweden; 2 School of Medical Sciences, Örebro University, Örebro, Sweden; 3 Department of Psychological and Brain Sciences, Indiana University, Bloomington, IN USA; 4 Institute of Neuroscience and Physiology, The Sahlgrenska Academy at Gothenburg University, Gothenburg, Sweden

**Keywords:** Schizophrenia, Bipolar disorder

## Abstract

Quasi-experimental and randomized controlled studies suggest that an enriched childhood rearing environment for at-risk individuals can reduce the risk for several psychiatric conditions. However, it remains uncertain if the reduced risk might be attributable to a general psychopathology factor common to all psychiatric conditions, versus specific psychopathology factors unique to only subsets of psychiatric conditions. In an at-risk sample, we estimated the association between an enriched childhood rearing environment and a latent bifactor model that captured both general and several specific psychopathology factors. The sample consisted of 881 full sibships where (a) the biological parents had (at least) one psychiatric diagnosis, suicide, or crime at any time in their lives, and (b) where (at least) one sibling was adopted away and raised by non-biological parents and (at least) one sibling raised by the biological parents. The exposure was whether a sibling was raised by biological versus adoptive parents. The outcome was a latent bifactor model based on nine conditions, including 7 in- or outpatient psychiatric diagnoses, suicide, and crimes. We recorded these outcomes from the birth of the siblings until the end of 2013, when the siblings were 34–64 years old. We used the marginal between-within model to estimate whether the adopted-away sibling(s) had lower scores on the latent factors. The latent bifactor model based on the nine conditions consisted of one general and three specific (externalizing, internalizing, and psychotic) psychopathology factors. The adopted-away siblings scored 0.27 (95% CI: −0.36, −0.18) standard deviations lower on the latent general psychopathology factor and 0.26 (95% CI: −0.38, −0.14) standard deviations lower on the latent specific externalizing factor, compared to their biological siblings who were raised by the biological parents. This result indicates that although genetics appears important for psychiatric comorbidity, the rearing environment also appears to play a systematic role in influencing the liability toward all mental health conditions among at-risk individuals. Improving the childhood rearing environment in high-risk families could potentially mitigate children’s liability toward all psychiatric conditions.

## Introduction

Observational studies show that children growing up in disadvantaged rearing environments (e.g., parents with psychiatric disorders or parents with low socioeconomic status) have elevated risks of adverse life outcomes, ranging from psychiatric disorders to criminal behavior and suicide [1–5]. Randomized controlled trials (RCTs) targeting disadvantaged families suggest that at least part of these associations are causal. For example, both the Perry Preschool Project (*N* = 123) and the Carolina Abecedarian Project (*N* = 114), which targeted African American children from families of low socioeconomic status, found that those randomized to early and lengthy childhood enrichment interventions displayed fewer criminal behaviors and depression symptoms by age 40 and 21, respectively [6–8]. Similarly, a meta-analysis based on 20 RCTs (pooled *N* = 2 689 children) found that treating parents with psychiatric disorders led to a 50% reduction in the offspring’s risk of mental disorders [9].

These RCTs, however, focused on only a few specific psychiatric outcomes at a time, even though comorbidity is the norm rather than the exception in psychiatry [10]. Therefore, it remains uncertain whether the effect is attributable to that which is common to all psychiatric conditions, versus that which is unique to subsets of psychiatric conditions (i.e., whether the effect is transdiagnostic or not). To remedy this, Wade and colleagues explored the effect of an enriched childhood rearing environment on a latent bifactor model, which partitions common parts of different psychiatric conditions into a general psychopathology factor and unique parts into several unrelated specific factors (e.g., a specific externalizing factor that captures variance related to substance misuse and anti-social tendencies not accounted for by the general factor) [11]. Among institutionalized 2-year-old children in Romania (*N* = 136), those randomized to high-quality foster care were rated 0.3 standard deviations lower on a general and a specific externalizing factor in adolescence, compared to those randomized to remain in institutionalized care. Whereas this implies that an enriched childhood rearing environment is causally linked to that which is common to all psychiatric conditions, this study did not follow the participants into adulthood and the sample size was relatively small such that the estimates were relatively imprecise.

Register-based observational studies offer the opportunity to study larger samples into adulthood. To isolate potential genetic confounding, past researchers have examined at-risk biological siblings who were discordant for adoption [12–16]. In Sweden, adoptive parents were carefully screened regarding their socioeconomic and health status, such that national adoptees in Sweden lived in, on average, a high-quality and stable rearing environment, which in turn seems to reduce the risk of psychiatric outcomes [12, 17, 18]. For instance, adopted-away individuals with biological parents with a life-time history of depression had a significantly reduced risk of major depression, compared to their biological siblings who remained with and were raised by the biological parents [12]. This protective effect of adoption was also found for alcohol use disorder [14] and criminal conviction [16] using the same quasi-experimental design. Similarly, individuals who had been in out-of-home care but were later adopted had significantly less psychiatric care and suicidal and criminal behaviors in adulthood, compared to their maternal (full- or half-) siblings who remained in out-of-home care throughout their childhood [19]. None of these studies, however, examined whether an enriched childhood rearing environment might have a transdiagnostic effect on all psychiatric conditions.

Using an at-risk sample of adult siblings discordant for adoption in early life, the goal of this study was to estimate the effect of an enriched childhood rearing environment, proxied via adoption, on a general psychopathology factor common to all psychiatric conditions and several specific psychopathology factors unique to subsets of psychiatric conditions.

## Method

### Sample

We created a population-based cohort study by linking several Swedish national registries using unique personal identification numbers, including the Total Population Register, the Multi-Generation Register, the National Patient Register, the National Crime Register, the Cause of Death Register, the Population and Housing Census, and the Longitudinal Integration Database for Health Insurance and Market Studies. A detailed description of these registries is shown in Supplementary Table 1.

We identified families in which (at least) one sibling was adopted away, whereas the other(s) remained with and were raised by the biological parents. In line with past research to identify at-risk children [12, 14, 16], we also required that at least one of the biological parents had at least one record of any psychiatric diagnoses, suicide (either suicide attempts or death by suicide), or crime (either court convictions or suspicion of violent or property crimes) at any time in their lives. We excluded siblings who were not born in Sweden, died (except for suicide), or emigrated before the end of the follow-up (December 31, 2013). To ensure that the children had lived with their respective parents from early on, we required that the adoptees had stayed in the same household as their adoptive parent for at least the first 10 years of their life, and, likewise, that the home-reared siblings had stayed in the same household as their biological parent for at least the first 10 years of their life. We used the apartment serial number from the Population and Housing Census (1960–1990 census) to identify whether the siblings were living in the same household as their parents. We excluded adoptees who later returned to live with their biological parents during their lifetime, and those who were adopted by their uncles/aunts.

The final sample consisted of 2 852 at-risk individuals from 881 full sibships, of which 970 were raised by adoptive parents and 1 882 remained with and were raised by their biological parents who had any psychiatric disorder, crime, or suicide. All children were born between 1950 and 1980 such that they were between 34 and 64 years old (mean age 52) at the end of follow-up (December 31, 2013).

### Exposure

The exposure was the rearing condition, that is, we compared individuals who were adopted away against their biological siblings who remained with and were raised by their biological parents. According to a Swedish regional study, common reasons for mothers to offer a child for adoption were being unmarried, having difficult financial circumstances, and lack of housing [20]. The adoptive parents tended to be older couples who had struggled with infertility and were carefully screened for their ability to provide a high-quality rearing environment (e.g., qualified occupations, high educational level, and good physical and mental health) [12, 17, 18, 20].

### Outcome

The outcome was a latent bifactor model estimated from the overlap (i.e., tetrachoric correlations) among nine observed outcomes. These included 7 psychiatric diagnoses (depression, anxiety [including obsessive-compulsive disorder], post-traumatic stress disorder [PTSD], alcohol-related conditions, drug-related conditions, bipolar disorder, and schizophrenia), crime (either court convictions or suspicion of violent or property crimes), and suicide (either suicide attempts or death by suicide). These were recorded from birth to the end of the follow-up. Supplementary Table 2 provides the International Classification of Diseases (ICD) codes, a description of convictions or suspicion classified as violent or property crimes, and the minimum cutoff age for each outcome (prior to which a diagnosis might be considered unreliable). All outcomes were treated as binary variables (i.e., ever recorded vs. not recorded).

### Statistical analyses

#### Association between childhood rearing condition and observed outcomes

First, to examine whether the childhood rearing environment was associated with the nine observed outcomes, we fitted a marginal logistic between-within model separately for each of the nine outcomes. The marginal between-within model estimates the exposure-outcome association within clusters (i.e., within clusters of biological siblings) by estimating a constant intercept common for all clusters and by including a cluster-mean exposure level, such that the model controls for unmeasured cluster-constant confounders (i.e., genetics shared between adopted-away and home-reared siblings) [21].

#### Latent bifactor model as outcome

Second, to estimate the effect of the childhood rearing environment on psychiatric comorbidity, we fitted a latent bifactor model to the nine observed outcomes. Latent bifactor models funnel the shared variance among all outcomes into a general factor, and several unrelated specific factors that capture covariation among subsets of outcomes not accounted for by the general factor [22]. These models are useful for examining whether a risk factor is associated with the common or unique parts of outcomes, and they are also estimated as free from measurement error variance. To derive the bifactor model, we fitted an exploratory factor analysis (EFA) that allowed all nine outcomes to load on all latent factors (i.e., cross-loadings were not constrained to zero). We decided on the number of factors to extract based on the scree plot, which contrasts the eigenvalues with the eigenvectors. We then rotated the extracted factors towards one general factor and several unrelated specific factors using a Direct Schmid-Leiman (DSL) rotation, a rank-deficient bifactor rotation [23].

#### Association between childhood rearing condition and latent bifactor model

Third, we fitted a marginal linear between-within model using the latent bifactor model as the outcome. That is, we regressed all latent factors simultaneously onto the exposure within clusters of biological siblings. We applied the DSL rotation within an Exploratory Structural Equation Model framework to estimate the associations between the childhood rearing environment and all latent factors [24, 25]. We used the robust maximum likelihood estimator and the sandwich estimator to estimate unbiased standard errors. In all models, we controlled for sibling sex and year of birth.

#### Sensitivity analyses

We conducted four sensitivity analyses. First, to examine whether similar results emerged by using different ways to capture psychiatric comorbidity while making fewer model assumptions, we created a total outcome sum score and extracted individual scores on the first principal component (PC1), which can be considered observed proxies of general psychopathology. The PC1 score was standardized to have a mean of 0 and a standard deviation of 1. We then fitted the marginal linear between-within model to the sum score and PC1. Second, to ensure that the results were not attributable to the number of latent factors extracted, we also extracted a 1- and 2-factor EFA. To test whether alternate ways of identifying the general and specific factors generated similar results, we also applied a non-rank deficient bifactor rotation [26]. Third, to account for potential cohort effects and to ensure that the home-reared sibling and the adopted-away sibling had roughly similar long follow-up periods, we excluded siblings who were born more than 8 years apart. Fourth, to test the robustness of the results for the nine observed outcomes when accounting for time to event, we followed the siblings until the time of the first record for outcomes, death, emigration, or the end of follow-up (December 31, 2013), whichever came first. We then used stratified Cox regression models with a separate stratum for each cluster of biological siblings.

Data management was conducted in SAS version 9.4. Marginal between-within models were performed with the gee function in the drgee package [27] using R version 4.3.1, and the bifactor models were identified using Mplus software [28]. The EFA (non-rank deficient) bifactor and the (rank-deficient) DSL rotation matrices were derived using the GPArotation package [29] in R.

## Results

Table 1 presents the distribution of characteristics of the home-reared siblings and adopted-away siblings and their biological parents and adoptive parents. As has been shown previously in Sweden [30] and the United States [15], compared to the biological parents, the adoptive parents displayed higher levels of education, lower rates of unemployment, and lower rates of social welfare recipiency.

**Table 1:** Description of sibling- and parent-specific characteristics for home-reared versus adopted-away siblings.

Sibling-specific characteristics N(%)	Home-Reared	Adopted-away
**N**	1882	970
**Sex**		
Male	951 (50.53)	459 (47.32)
Female	931 (49.47)	511 (52.68)
**Birth year**		
1950–1960	978 (51.97)	392 (40.41)
1960–1970	751 (39.90)	492 (50.72)
1970–1980	153 (8.13)	86 (8.87)
**Parent-specific characteristics N(%)**		
**N**^**a**^	881	942
**Highest educational level**		
Compulsory school ( ≤ 9 years)	418 (47.45)	337 (35.77)
Upper secondary school	392 (44.49)	377 (40.02)
University	67 (7.60)	209 (22.19)
Missing	4 (0.45)	19 (2.02)
**Ever received social welfare**		
No	440 (49.94)	815 (86.52)
Yes	429 (48.69)	102 (10.83)
Missing	12 (1.36)	25 (2.65)
**Long-term unemployment**^**b**^		
No	728 (82.63)	847 (89.92)
Yes	135 (15.32)	63 (6.69)
Missing	18 (2.04)	32 (3.40)
**Any outcome**^**c**^		
No	0 (0.00)	582 (61.78)
Yes	1 (100.00)	360 (38.22)
**Any psychiatric disorder**		
No	160 (18.16)	629 (66.77)
Yes	721 (81.84)	313 (33.23)
**Suicide**		
No	687 (77.98)	895 (95.01)
Yes	194 (22.02)	47 (4.99)
**Crime**		
No	502 (56.98)	885 (93.95)
Yes	379 (43.02)	57 (6.05)

N^a^ is the number of pairs for biological parents and adoptive parents.

^b^Long-term unemployment was defined as an unemployment status of one year or more.

^c^Included any psychiatric disorders, suicide, and crime.

### Associations between childhood rearing condition and observed outcomes

Table 2 presents the regression results between the childhood rearing condition and each of the nine observed outcomes. Compared to the home-reared siblings, the adopted-away siblings had a significantly lower risk of developing depression (OR = 0.58 95% CI: 0.43, 0.78), anxiety (OR = 0.69 95% CI: 0.52, 0.92), PTSD (OR = 0.68 95% CI: 0.47, 0.99), drug-related conditions (OR = 0.53 95% CI: 0.38, 0.76), being convicted or suspected of crime (OR = 0.57 95% CI: 0.47, 0.68), and being diagnosed with or dying by suicide (OR = 0.69 95% CI: 0.52, 0.92). The adopted-away siblings also displayed a lower risk for bipolar disorder, schizophrenia, or alcohol-related conditions, but these associations were not statistically different from the null.

**Table 2:** Associations between childhood rearing condition (adopted-away versus home-reared siblings) and nine observed outcomes.

Sibling outcomes	Odds ratio (95% CI)*	*p* value
Depression	0.58 (0.43, 0.78)	<0.001
Anxiety	0.69 (0.52, 0.92)	0.012
PTSD	0.68 (0.47, 0.99)	0.042
Suicide	0.69 (0.52, 0.92)	0.012
Alcohol-related conditions	0.76 (0.58, 1.00)	0.054
Drug-related conditions	0.53 (0.38, 0.76)	0.001
Crime	0.57 (0.47, 0.68)	<0.001
Bipolar disorder	0.75 (0.42, 1.36)	0.347
Schizophrenia	0.53 (0.25, 1.13)	0.099

Associations were adjusted for sibling sex and year of birth.

*PTSD* post-traumatic stress disorder.

### Latent bifactor model as outcome

The first five eigenvalues of the nine outcomes were 4.58, 1.25, 0.90, 0.57, and 0.46, so we decided to extract three exploratory factors. We rotated the three extracted factors toward a bifactor model consisting of one general (loading range: 0.43–0.64), and three specific factors capturing psychotic, externalizing, and internalizing conditions, respectively (Table 3).

**Table 3:** Factor loadings of exploratory factor analysis based on nine observed outcomes.

	Direct Schmid-Leiman bifactor rotation			
Sibling outcomes	General factor	Internalizing factor	Externalizing factor	Psychotic factor
Depression	**0.62**	**0.65**	0.03	−0.01
Anxiety	**0.54**	**0.55**	0.06	−0.08
PTSD	**0.52**	**0.54**	0.05	−0.14
Suicide	**0.60**	**0.33**	0.27	0.14
Alcohol-related conditions	**0.62**	0.17	**0.47**	0.05
Drug-related conditions	**0.64**	0.10	**0.54**	0.09
Crime	**0.43**	−0.12	**0.55**	0.04
Bipolar disorder	**0.51**	**0.33**	0.07	**0.66**
Schizophrenia	**0.54**	0.24	0.25	**0.31**

Factor loadings greater than 0.30 are bolded for visual clarity.

*PTSD* post-traumatic stress disorder.

### Association between childhood rearing condition and latent bifactor model

Table 4 displays the regression results between the childhood rearing condition and the latent bifactor model. Adopted-away siblings scored 0.27 (95% CI: −0.36, −0.18) standard deviations lower on the general psychopathology factor, compared to their biological siblings who remained with and were raised by the biological parents. In addition, the adopted-away siblings also scored 0.26 (95% CI: −0.38, −0.14) standard deviations lower on the specific externalizing factor. There were no significant associations between childhood rearing condition and the specific internalizing or the specific psychotic factors.

**Table 4:** Associations between childhood rearing condition (adopted-away versus home-reared siblings) and latent outcomes.

Latent outcomes	Beta (95% CI)	*p* value
General factor	−0.27 (−0.36, −0.18)	<0.001
Internalizing factor	−0.04 (−0.16, 0.08)	0.539
Externalizing factor	−0.26 (−0.38, −0.14)	<0.001
Psychotic factor	0.12 (−0.15, 0.39)	0.394

Latent outcomes were identified via a Direct Schmid-Leiman rotated EFA of the nine observed outcomes. All latent factors were standardized to unity. Associations are adjusted for sibling sex and year of birth.

### Results from Sensitivity Analyses

First, adopted-away siblings scored on average 0.30 (95% CI: −0.39, −0.21) units lower on the outcome sum score, and 0.21 (95% CI: −0.28, −0.13) standard deviations lower on the PC1 of the outcomes, compared to their full siblings who remained with and were raised by the biological parents. Second, the factor loadings of the 1-factor EFA, (DSL-rotated) 2-factor EFA, and (non-rank deficient orthogonal bifactor-rotated) 3-factor EFA, are shown in Supplementary Table 3. Consistent with the main results for the latent outcomes, adopted-away siblings had lower scores on the latent general psychopathology and the specific externalizing factors, but not on the specific internalizing factor, compared to their home-reared siblings, regardless of the number of factors extracted or type of bifactor rotation used (Supplementary Table 4). Third, when restricting the age difference between home-reared and adopted-away siblings to 8 years or less, the results were similar to the results for the nine observed outcomes and the latent outcomes (Supplementary Table 5). Fourth, when taking the time to event into account, the results were consistent with the results that used logistic regression for the nine observed outcomes, with the exception that a statistically significant association was additionally observed for alcohol-related conditions (Supplementary Table 6).

## Discussion

We observed that individuals raised by adoptive parents – who had higher levels of education, lower rates of unemployment, and lower rates of social welfare recipiency – had a lower risk of developing broad psychiatric comorbidity, compared to their biological siblings who remained with and were raised by the biological parents with psychiatric conditions. In addition, the adopted-away siblings also scored lower on a specific externalizing factor. These results extend a previous RCT, which found similar protective effects of an enriched childhood rearing environment on general and externalizing psychopathology factors in adolescence, into middle adulthood with a larger sample size [11]. This implies that although family, twin, and genomic studies have highlighted the importance of genetic contributions to psychiatric comorbidity [31–34], the rearing environment also appears to play a systematic role in influencing the liability toward all mental health conditions among at-risk individuals.

Early interventions aimed at enriching the childhood rearing environment, such as treating parental mental disorders or improving the socio-economic status, in high-risk families might reduce children’s transdiagnostic liability toward psychiatric comorbidity in adulthood. The broader impact of reducing transdiagnostic liability may draw greater attention from public health policymakers. In particular, transdiagnostic interventions aiming to address multiple simultaneous difficulties with universal therapeutic protocols may prove especially beneficial for children growing up in high-risk families or those predisposed to mental health issues, as evidenced by their cost-effectiveness, generalizability, and increasing popularity [35–37].

Because it remains uncertain what aspect(s) of the adoptive parents might function to protect the children from psychiatric comorbidity, we review three possibilities. First, past quasi-causal studies have highlighted the importance of financial resources. Based on an adoption design that ruled out genetic confounding, children (*N* = 5698) raised by richer (vs. poorer) Norwegian non-biological (i.e., adoptive) parents had fewer psychiatric diagnoses [3]. Similarly, tribe members (*N* = 1266) who received annual cash transfers throughout their childhood from a Native American-run local casino had fewer mental health problems 30 years later, compared to non-tribe peers (who did not receive cash transfers) [38]. This protective effect of socioeconomic advantages might stem from the ability of parents to make greater material and psychological investments in their children, thereby reducing the risk of adverse psychiatric outcomes [39, 40].

A second possibility is that the protective effect was primarily attributable to being raised by parents with fewer psychiatric problems. This hypothesis is supported by evidence that the beneficial effect of being raised by adoptive parents disappeared when the adoptive parents or stepsiblings in the adoptive home had depression [12], that the association between parental and offspring psychiatric conditions tends to remain when using different genetically informative designs [41–43], and that treatment of parental psychiatric disorders, in turn, reduces the offspring’s risk of developing mental health problems [9].

Third, the protective effect might be attributable to improved parenting skills. Whereas the high-quality, intense, and costly early childhood interventions in the Perry Preschool Project and the Carolina Abecedarian Project primarily focused on improving children’s ability to plan, execute, and evaluate tasks, they also included a smaller parent training component. A recent review showed that programs that included only the less costly and briefer parent training component generated comparable and equally sustained protective effects [44]. One speculation might be that effective parents teach children skills that promote executive functions that in turn have a transdiagnostic protective effect on all psychiatric conditions as captured via a general psychopathology factor [45–47], and a suitable degree of impulse control and restraint that accounts for the association with the specific externalizing factor. Potentially supporting this speculation, data from 118 sets of adoption-linked families in the United States showed that adoptive mothers displayed more guidance and less harsh parenting compared to the adoptee’s biological mothers [15].

### Limitations

Our results should be interpreted with several limitations in mind. First, in contrast to an RCT, the sibling comparison design cannot control for unmeasured confounders that siblings do not share. Second, the sibling comparison design inherently limits analysis to candidate environments that vary among siblings, thereby excluding families with a single child, all adopted-away children, or no adopted-away children. This raises concerns about the representativeness of our sample. Nevertheless, although our results might not generalize to all families, at-risk families are of interest for clinical and intervention purposes. Third, the adoption of one or more siblings might have detrimental effects on the mental health of the home-reared siblings, or cause trauma for the biological parents, which in turn could affect their parenting of the other children. Fourth, potential bias may arise if adopted-away siblings maintain substantial contact with their biological parents. While the frequency of such contact was not ascertainable, we excluded individuals who later returned to live with their biological parents during their lifetime. Fifth, because a thorough medical examination of the adoptee and a matching process of adoptees and adoptive parents must take place prior to the adoption [18], adoptions may not be random, potentially introducing bias into the results.

## Conclusion

At-risk individuals who were raised in adoptive families, which displayed higher socio-economic status, had lower scores on a general factor common to all psychiatric conditions in middle adulthood, compared to their full siblings who remained with and were raised by biological parents with a life-time history of one or more psychiatric conditions. Interventions aimed at enriching the childhood rearing environment in high-risk families might reduce children’s transdiagnostic liability toward psychiatric comorbidity in adulthood.

### Data sharing

The Public Access to Information and Secrecy Act in Sweden prohibits us from making individual-level data publicly available. Researchers can apply for individual-level data through Statistics Sweden at: https://www.scb.se/en/services/guidancefor-researchers-and-universities/.

## Supplementary information

Supplement

## Data Availability

Code used for these analyses are available from the corresponding author upon reasonable request.

## References

[1] Uher R, Pavlova B, Radua J, Provenzani U, Najafi S, Fortea L, et al. Transdiagnostic risk of mental disorders in offspring of affected parents: a meta-analysis of family high-risk and registry studies. World Psychiatry. 2023;22:433–48. 37713573 10.1002/wps.21147 PMC10503921

[2] Goodday SM, Shuldiner J, Bondy S, Rhodes AE. Exposure to parental psychopathology and offspring’s risk of suicide-related thoughts and behaviours: a systematic review. Epidemiol Psychiatr Sci. 2019;28:179–90. 28748774 10.1017/S2045796017000397 PMC6998933

[3] Kinge JM, Overland S, Flato M, Dieleman J, Rogeberg O, Magnus MC, et al. Parental income and mental disorders in children and adolescents: prospective register-based study. Int J Epidemiol. 2021;50:1615–27. 33975355 10.1093/ije/dyab066 PMC8580274

[4] Najman JM, Hayatbakhsh MR, Clavarino A, Bor W, O’Callaghan MJ, Williams GM. Family poverty over the early life course and recurrent adolescent and young adult anxiety and depression: a longitudinal study. Am J Public Health. 2010;100:1719–23. 20634459 10.2105/AJPH.2009.180943 PMC2920957

[5] McLaughlin KA, Greif Green J, Gruber MJ, Sampson NA, Zaslavsky AM, Kessler RC. Childhood adversities and first onset of psychiatric disorders in a national sample of US adolescents. Arch Gen Psychiatry. 2012;69:1151–60. 23117636 10.1001/archgenpsychiatry.2011.2277 PMC3490224

[6] Conti G, Heckman J, Pinto R. The effects of two influential early childhood interventions on health and healthy behaviour. Econ J. 2016;126:F28–F65. 10.1111/ecoj.12420 PMC5331750 28260805

[7] McLaughlin AE, Campbell FA, Pungello EP, Skinner M. Depressive symptoms in young adults: the influences of the early home environment and early educational child care. Child Dev. 2007;78:746–56. 17517002 10.1111/j.1467-8624.2007.01030.x

[8] Belfield CR, Nores M, Barnett S, Schweinhart L. The high/scope perry preschool program - cost-benefit analysis using data from the age-40 Followup. J Hum Resour. 2006;41:162–90.

[9] Lannes A, Bui E, Arnaud C, Raynaud JP, Revet A. Preventive interventions in offspring of parents with mental illness: a systematic review and meta-analysis of randomized controlled trials. Psychol Med. 2021;51:2321–36. 34435556 10.1017/S0033291721003366

[10] Plana-Ripoll O, Pedersen CB, Holtz Y, Benros ME, Dalsgaard S, de Jonge P, et al. Exploring comorbidity within mental disorders among a Danish national population. JAMA Psychiatry. 2019;76:259–70. 30649197 10.1001/jamapsychiatry.2018.3658 PMC6439836

[11] Wade M, Fox NA, Zeanah CH, Nelson CA. Effect of foster care intervention on trajectories of general and specific psychopathology among children with histories of institutional rearing: a randomized clinical trial. JAMA Psychiatry. 2018;75:1137–45. 30267045 10.1001/jamapsychiatry.2018.2556 PMC6248099

[12] Kendler KS, Ohlsson H, Sundquist J, Sundquist K. The rearing environment and risk for major depression: a Swedish national high-risk home-reared and adopted-away co-sibling control study. Am J Psychiatry. 2020;177:447–53. 32340466 10.1176/appi.ajp.2019.19090911 PMC10916706

[13] Kendler KS, Turkheimer E, Ohlsson H, Sundquist J, Sundquist K. Family environment and the malleability of cognitive ability: a Swedish national home-reared and adopted-away cosibling control study. Proc Natl Acad Sci USA. 2015;112:4612–7. 25831538 10.1073/pnas.1417106112 PMC4403216

[14] Kendler KS, Ohlsson H, Sundquist J, Sundquist K. The rearing environment and the risk for alcohol use disorder: a Swedish national high-risk home-reared v. adopted co-sibling control study. Psychol Med. 2021;51:2370–7. 32317035 10.1017/S0033291720000963 PMC7870033

[15] Natsuaki MN, Neiderhiser JM, Harold GT, Shaw DS, Reiss D, Leve LD. Siblings reared apart: a sibling comparison study on rearing environment differences. Dev Psychol. 2019;55:1182–90. 30816723 10.1037/dev0000710 PMC6533126

[16] Kendler KS, Morris NA, Ohlsson H, Lönn SL, Sundquist J, Sundquist K. Criminal offending and the family environment: Swedish national high-risk home-reared and adopted-away co-sibling control study. Br J Psychiatry. 2016;209:294–9. 27539295 10.1192/bjp.bp.114.159558

[17] Hamilton L, Cheng S, Powell B. Adoptive parents, adaptive parents: evaluating the importance of biological ties for parental investment. Am Sociol Rev. 2016;72:95–116.

[18] Björklund A, Lindahl M, Plug E Intergenerational effects in Sweden: what can we learn from adoption data? SSRN Electron J. 2004. 10.2139/ssrn.565401

[19] Hjern A, Vinnerljung B, Brännström L. Outcomes in adulthood of adoption after long-term foster care: a sibling study. Dev Child Welf. 2019;1:61–75. 10.1177/1077559519898755 31960707

[20] Bohman M. A study of adopted children, their back-ground, environment and adjustment. Acta Paediatr Scand. 1972;61:90–7. 5062971 10.1111/j.1651-2227.1972.tb15907.x

[21] Sjölander A. Estimation of marginal causal effects in the presence of confounding by cluster. Biostatistics. 2021;22:598–612. 31804668 10.1093/biostatistics/kxz054

[22] Lahey BB, Moore TM, Kaczkurkin AN, Zald DH. Hierarchical models of psychopathology: empirical support, implications, and remaining issues. World Psychiatry. 2021;20:57–63. 33432749 10.1002/wps.20824 PMC7801849

[23] Waller NG. Direct schmid-leiman transformations and rank-deficient loadings matrices. Psychometrika. 2018;83:858–70. 29204802 10.1007/s11336-017-9599-0

[24] Chen C, Chang Z, Kuja-Halkola R, D’Onofrio BM, Larsson H, Andell P, et al. Associations between general and specific mental health conditions in Young adulthood and cardiometabolic complications in middle adulthood: a 40-year longitudinal familial coaggregation study of 672,823 Swedish individuals. Am J Psychiatry. 2024;181:651–7. 38263878 10.1176/appi.ajp.20220951

[25] Asparouhov T, Muthén B. Exploratory structural equation modeling. Struct Equ Model. 2009;16:397–438.

[26] Yung Y-F, Thissen D, McLeod LD. On the relationship between the higher-order factor model and the hierarchical factor model. Psychometrika. 1999;64:113–28.

[27] Zetterqvist J, Sjölander A. Doubly robust estimation with the R package drgee. Epidemiol Methods. 2015;4:69–86.

[28] Muthén LK, Muthén BO Mplus user’s guide. Seventh Edition. Los Angeles, CA: Muthén & Muthén; 2012. https://www.statmodel.com/download/usersguide/MplusUserGuideVer_7.pdf.

[29] Bernaards CA, Jennrich RI. Gradient projection algorithms and software for arbitrary rotation criteria in factor analysis. Educ Psychol Meas. 2016;65:676–96.

[30] Kendler KS, Sundquist K, Ohlsson H, Palmer K, Maes H, Winkleby MA, et al. Genetic and familial environmental influences on the risk for drug abuse: a national Swedish adoption study. Arch Gen Psychiatry. 2012;69:690–7. 22393206 10.1001/archgenpsychiatry.2011.2112 PMC3556483

[31] Pettersson E, Larsson H, Lichtenstein P. Common psychiatric disorders share the same genetic origin: a multivariate sibling study of the Swedish population. Mol Psychiatry. 2016;21:717–21. 26303662 10.1038/mp.2015.116

[32] Lahey BB, Van Hulle CA, Singh AL, Waldman ID, Rathouz PJ. Higher-order genetic and environmental structure of prevalent forms of child and adolescent psychopathology. Arch Gen Psychiatry. 2011;68:181–9. 21300945 10.1001/archgenpsychiatry.2010.192 PMC3322461

[33] Allegrini AG, Cheesman R, Rimfeld K, Selzam S, Pingault JB, Eley TC, et al. The p factor: genetic analyses support a general dimension of psychopathology in childhood and adolescence. J Child Psychol Psychiatry. 2020;61:30–9. 31541466 10.1111/jcpp.13113 PMC6906245

[34] Neumann A, Nolte IM, Pappa I, Ahluwalia TS, Pettersson E, Rodriguez A, et al. A genome-wide association study of total child psychiatric problems scores. PLoS one. 2022;17:e0273116. 35994476 10.1371/journal.pone.0273116 PMC9394806

[35] Dozois DJA, Seeds PM, Collins KA. Transdiagnostic approaches to the prevention of depression and anxiety. J Cogn Psychother. 2009;23:44–59.

[36] Wang P, Wang Z, Qiu S. Universal, school-based transdiagnostic interventions to promote mental health and emotional wellbeing: a systematic review. Child Adolesc Psychiatry Ment Health. 2024;18:47. 38600562 10.1186/s13034-024-00735-x PMC11007989

[37] Garcia-Lopez L-J, Jimenez-Vazquez D, Muela-Martinez J-A, Piqueras JA, Espinosa-Fernandez L, Canals-Sans J, et al. Effectiveness of a transdiagnostic indicated preventive intervention for adolescents at high risk for anxiety and depressive disorders. Curr Psychol. 2023;43:15484–98.

[38] Copeland WE, Tong G, Gaydosh L, Hill SN, Godwin J, Shanahan L, et al. Long-term outcomes of childhood family income supplements on adult functioning. JAMA Pediatr. 2022;176:1020–6. 35994270 10.1001/jamapediatrics.2022.2946 PMC9396462

[39] Conger RD, Donnellan MB. An interactionist perspective on the socioeconomic context of human development. Annu Rev Psychol. 2007;58:175–99. 16903807 10.1146/annurev.psych.58.110405.085551

[40] Bradley RH, Corwyn RF. Socioeconomic status and child development. Annu Rev Psychol. 2002;53:371–99. 11752490 10.1146/annurev.psych.53.100901.135233

[41] McAdams TA, Neiderhiser JM, Rijsdijk FV, Narusyte J, Lichtenstein P, Eley TC. Accounting for genetic and environmental confounds in associations between parent and child characteristics: a systematic review of children-of-twins studies. Psychol Bull. 2014;140:1138–73. 24749497 10.1037/a0036416

[42] D’Onofrio BM, Turkheimer EN, Eaves LJ, Corey LA, Berg K, Solaas MH, et al. The role of the children of twins design in elucidating causal relations between parent characteristics and child outcomes. J Child Psychol Psychiatry. 2003;44:1130–44. 14626455 10.1111/1469-7610.00196

[43] Jami ES, Hammerschlag AR, Bartels M, Middeldorp CM. Parental characteristics and offspring mental health and related outcomes: a systematic review of genetically informative literature. Transl Psychiatry. 2021;11:197. 33795643 10.1038/s41398-021-01300-2 PMC8016911

[44] Garcia JL, Heckman JJ. Parenting promotes social mobility within and across generations. Annu Rev Econ. 2023;15:349–88. 10.1146/annurev-economics-021423-031905 PMC10972614 38545330

[45] Snyder HR, Miyake A, Hankin BL. Advancing understanding of executive function impairments and psychopathology: bridging the gap between clinical and cognitive approaches. Front Psychol. 2015;6:328. 25859234 10.3389/fpsyg.2015.00328 PMC4374537

[46] Caspi A, Moffitt TE. All for one and one for all: mental disorders in one dimension. Am J Psychiatry. 2018;175:831–44. 29621902 10.1176/appi.ajp.2018.17121383 PMC6120790

[47] Harden KP, Engelhardt LE, Mann FD, Patterson MW, Grotzinger AD, Savicki SL, et al. Genetic associations between executive functions and a general factor of psychopathology. J Am Acad Child Adolesc Psychiatry. 2020;59:749–58. 31102652 10.1016/j.jaac.2019.05.006 PMC6986791

