# Supplementary material for: Association between the childhood rearing environment and general and specific psychopathology factors in middle adulthood: a Swedish National High-Risk Home-Reared versus Adopted-Away Sibling Comparison Study
Source: Mol Psychiatry. 2025 Apr 1;30(9):4023–8. doi: 10.1038/s41380-025-02979-1 (PMC12339380; doi:10.1038/s41380-025-02979-1)
Supplement: Supplementary file 1 — Supplement [file 41380_2025_2979_MOESM1_ESM.pdf]

Supplementary Online Content

Article title

Association between the Childhood Rearing Environment and General and Specific Psychopathology Factors in Middle Adulthood: A Swedish National High-Risk Home-Reared versus Adopted-Away Sibling Comparison Study

Authors

Mengping Zhou MSc[1], Henrik Larsson PhD[1,2], Brian M. D'Onofrio PhD[1,3], Mikael Landén PhD[1,4], Paul Lichtenstein PhD[1], Erik Pettersson PhD[1]

[1] Department of Medical Epidemiology and Biostatistics, Karolinska Institutet, Stockholm, Sweden

[2] School of Medical Sciences, Örebro University, Örebro, Sweden

[3] Department of Psychological and Brain Sciences, Indiana University, Bloomington, IN, USA

[4] Institute of Neuroscience and Physiology, The Sahlgrenska Academy at Gothenburg University, Gothenburg, Sweden

Supplementary Content

Supplementary Table 1. Description of registries and variables extracted

Supplementary Table 2. The ICD code, classified convictions or suspicion for crimes, and the cut-off age for each outcome

Supplementary Table 3. Factor loadings for sensitivity analyses of different bifactor models

Supplementary Table 4. Sensitivity analyses for sibling latent outcomes when using different bifactor models

Supplementary Table 5. Sensitivity analyses for sibling outcomes when restricting the age difference between home-reared and adopted-away siblings to 8 years or less

Supplementary Table 6. Sensitivity analyses for sibling observed outcomes when using Cox regression

Supplementary Table 1. Description of registries and variables extracted

Register	Description	Variables
Total Population Register	Established in 1968 and includes demographic information (e.g., sex, age, place of birth) for the entire Swedish population.[1]	Individual identification number, birthyear, sex
Multi-Generation Register	Links all index persons born in Sweden since 1932 and alive in 1960 to their biological or adoptive parent.[2]	Individual identification number
National Patient Register	Includes individual-based records of psychiatric inpatient care since 1973 (complete since 1987) and psychiatric outpatient care since 2001 (complete since 2010). All diagnoses were recorded according to the International Classification of Diseases (ICD); 8th (1973/1969-1986), 9th (1987-1996), and 10th (1997-2009) revision.[3,4]	Disorders, suicide attempts
National Crime Register	Comprises all registered criminal convictions or suspicion of those aged 15 and older (the age of criminal responsibility) since 1973.[5]	Court convictions and suspicion of violent and property crimes
Cause of Death Register	Records all deaths in Sweden since 1952 and provides information on causes of death according to ICD.[6]	Death by suicide
Population and Housing Census	Information about employment, households, and accommodation, has been carried out every fifth year since 1960.	Apartment serial number
Longitudinal Integration Database for Health Insurance and Market Studies	Information from the labor market and educational and social sectors for all individuals registered in Sweden over 16 years of age since 1990.[7]	Social welfare recipiency, unemployment, parental educational level

Supplementary Table 2. The ICD code, classified convictions or suspicion for crimes, and the cut-off age for each outcome

Exposure/outcome	ICD 08 (1969-1986)	ICD 09 (1987-1996)	ICD 10 (1997-)	Minimum age
Schizophrenia	295.0-295.4, 295.6, 295,7, 295.8, 295.9	295A-295E, 295G, 295H, 295W, 295X	F20, F25	15
Bipolar disorder	296.0-296.3, 296.8, 296.9	296A-296E, 296W, 296X	F30, F31	15
Depression	300.4	311, 300E	F32-F39	5
Anxiety (including obsessive-compulsive disorder)	300 (except 300.4)	300, (except 300E)	F40-F42, F44-F45, F48	5
Post-traumatic stress disorder	307	308, 309	F43	2
Alcohol-related conditions	291, 303	291, 303, 305A	F10 (except F10.5)	12
Drug-related conditions	304	292, 304, 305X	F11-F19 (except F1x.5)	12
Suicide (either suicide attempts or death by suicide)	E950-959 E980-989	E950-959 E980-989	X60-X84, Y10-Y34	10
Crime (either court convictions or suspicion of violent or property crimes)	homicide; assault; robbery; threats and violence against an officer; gross violation of a person's/woman's integrity; unlawful coercion; unlawful threats; kidnapping; illegal confinement; arson; intimidation; sexual offence (excluding prostitution and the buying of sexual services but including child pornography), property crimes			15

Supplementary Table 3. Factor loadings for sensitivity analyses of different bifactor models

	1-factor EFA	(DSL-rotated) 2-factor EFA			(orthogonal bifactor-rotated) 3-factor EFA		
**Offspring outcomes**	**General factor**	**General factor**	**Internalizing factor**	**Externalizing factor**	**General factor**	**Internalizing factor**	**Unspecified factor**
Depression	**0.78**	**0.66**	**0.61**	0.05	**0.62**	**0.65**	0.08
Anxiety	**0.71**	**0.58**	**0.51**	0.07	**0.53**	**0.57**	-0.02
PTSD	**0.70**	**0.56**	**0.51**	0.04	**0.49**	**0.59**	-0.05
Suicide	**0.75**	**0.60**	0.28	**0.32**	**0.71**	0.23	-0.03
Alcohol-related conditions	**0.75**	**0.61**	0.12	**0.49**	**0.74**	0.10	-0.27
Drug-related conditions	**0.78**	**0.63**	0.07	**0.56**	**0.80**	0.01	-0.30
Crime	**0.45**	**0.40**	-0.16	**0.56**	**0.57**	-0.19	-0.37
Bipolar disorder	**0.68**	**0.55**	0.28	0.27	**0.76**	0.03	**0.49**
Schizophrenia	**0.66**	**0.52**	0.12	**0.40**	**0.70**	0.08	0.09

Note. DSL = Direct Schmid-Leiman. PTSD = post-traumatic stress disorder. Factor loadings greater than 0.30 are bolded for visual clarity.

Supplementary Table 4. Sensitivity analyses for sibling latent outcomes when using different bifactor models

Model	Latent outcomes	Beta (95% CI)	*p* value
EFA 1 factor	General factor	-0.34 (-0.45, -0.23)	<0.001
(DSL-rotated) 2-factor EFA	General factor	-0.26 (-0.36, -0.17)	<0.001
	Internalizing factor	-0.03 (-0.15, 0.09)	0.582
	Externalizing factor	-0.23 (-0.34, -0.12)	<0.001
(orthogonal bifactor-rotated) 3-factor EFA	General factor	-0.27 (-0.41, -0.12)	<0.001
	Internalizing factor	-0.06 (-0.24, 0.12)	0.501
	Unspecified factor	0.28 (0.05, 0.52)	0.017

Note. DSL = Direct Schmid-Leiman. Associations were adjusted for sibling sex and year of birth.

Supplementary Table 5. Sensitivity analyses for sibling outcomes when restricting the age difference between home-reared and adopted-away siblings to 8 years or less

Sibling outcomes		Estimator* (95% CI)	*p* value
Observed outcomes	Depression	0.56 (0.41, 0.76)	<0.001
	Anxiety	0.61 (0.45, 0.83)	0.002
	PTSD	0.69 (0.46, 1.02)	0.062
	Suicide	0.63 (0.47, 0.86)	0.003
	Alcohol-related conditions	0.76 (0.58, 1.01)	0.055
	Drug-related conditions	0.56 (0.39, 0.80)	0.001
	Crime	0.61 (0.49, 0.76)	<0.001
	Bipolar disorder	0.69 (0.37, 1.29)	0.246
	Schizophrenia	0.51 (0.24, 1.08)	0.079
Latent outcomes	General factor	-0.28 (-0.37, -0.20)	<0.001
	Internalizing factor	-0.08 (-0.20, 0.04)	0.182
	Externalizing factor	-0.21 (-0.33, -0.10)	<0.001
	Psychotic factor	0.00 (-0.32, 0.31)	0.985

Note. Latent outcomes were identified via a Direct Schmid-Leiman rotated EFA of the nine observed outcomes. All latent outcomes were standardized to unity. *The estimator is the odds ratio for observed outcomes and beta for latent outcomes. Associations were adjusted for sibling sex and year of birth. PTSD = post-traumatic stress disorder.

Supplementary Table 6. Sensitivity analyses for sibling observed outcomes when using Cox regression

**Sibling outcome**	**HR (95% CI)**	***p* value**
Depression	0.58 (0.46, 0.74)	<0.001
Anxiety	0.63 (0.49, 0.81)	<0.001
PTSD	0.69 (0.50, 0.95)	0.023
Suicide	0.69 (0.55, 0.87)	0.002
Alcohol-related conditions	0.68 (0.55, 0.85)	0.001
Drug-related conditions	0.51 (0.38, 0.68)	<0.001
Crime	0.59 (0.52, 0.68)	<0.001
Bipolar disorder	0.62 (0.37, 1.04)	0.071
Schizophrenia	0.75 (0.37, 1.53)	0.426

Note. Associations were adjusted for sibling sex and year of birth. PTSD = post-traumatic stress disorder.

Reference

1. Ludvigsson JF, Almqvist C, Bonamy A-KE, et al. Registers of the Swedish total population and their use in medical research. *European Journal of Epidemiology*. 2016-02-01 2016;31(2):125-136. doi:10.1007/s10654-016-0117-y

2. Ekbom A. The Swedish Multi-generation Register. *Methods in Molecular Biology*. Humana Press; 2011:215-220.

3. Socialstyrelsen. The National Patient Register. Accessed 05.30, 2022. https://www.socialstyrelsen.se/en/statistics-and-data/registers/national-patient-register/

4. Ludvigsson JF, Andersson E, Ekbom A, et al. External review and validation of the Swedish national inpatient register. *BMC Public Health*. Jun 9 2011;11(1):450. doi:10.1186/1471-2458-11-450

5. Frisell T, Lichtenstein P, Långström N. Violent crime runs in families: a total population study of 12.5 million individuals. *Psychological Medicine*. 2011-01-01 2011;41(1):97-105. doi:10.1017/s0033291710000462

6. Brooke HL, Talbäck M, Hörnblad J, et al. The Swedish cause of death register. *European Journal of Epidemiology*. 2017-09-01 2017;32(9):765-773. doi:10.1007/s10654-017-0316-1

7. Ludvigsson JF, Svedberg P, Olén O, Bruze G, Neovius M. The longitudinal integrated database for health insurance and labour market studies (LISA) and its use in medical research. *European Journal of Epidemiology*. 2019-04-01 2019;34(4):423-437. doi:10.1007/s10654-019-00511-8
